# Efficacy of Clomiphene Citrate Versus Enclomiphene Citrate for Male Infertility Treatment: A Retrospective Study

**DOI:** 10.7759/cureus.41476

**Published:** 2023-07-06

**Authors:** Jamie Thomas, Maria Camila Suarez Arbelaez, Manish Narasimman, Alexander R Weber, Ruben Blachman-Braun, Joshua T White, Braian Ledesma, Armin Ghomeshi, Miguel A Jara-Palacios, Ranjith Ramasamy

**Affiliations:** 1 Desai Sethi Urology Institute, University of Miami Miller School of Medicine, Miami, USA; 2 Desai Sethi Urology Institute, University of Miami Miller School of Medicine/Jackson Memorial Hospital, Miami, USA; 3 Urology, Florida International University/Herbert Wertheim College of Medicine, Miami, USA; 4 Endocrinology, University of Miami Miller School of Medicine, Miami, USA

**Keywords:** gonadotropins, sperm, anabolic steroid abuse, male factor infertility, hypogonadism, enclomiphene citrate, clomiphene citrate

## Abstract

Introduction

Infertility and hypogonadism in males can greatly affect their reproductive health and overall well-being. Since exogenous testosterone administration for hypogonadism management may disrupt the normal hormonal cascade necessary for spermatogenesis, clomiphene citrate (CC) and enclomiphene citrate (EC) are medications often used to manage hypogonadism and male infertility. This study aims to directly compare the effects of CC and EC on serum testosterone levels and semen parameters in men to determine which medication may have an advantage in managing these conditions.

Materials and methods

We retrospectively analyzed ≥18-year-old men presenting with primary infertility, abnormal semen parameters, or hypogonadism who received CC or EC monotherapy for at least three months between January 2021 and December 2022. We compared baseline and follow-up hormone levels, semen parameters, and demographics. Variables were compared using paired and unpaired t-tests. Significance was assessed at *p*<0.05.

Results

A total of 46 men received EC and 32 men received CC. The median age was 42 (IQR: 34-47.75) years in men who received EC and 41 (IQR: 36-44) years in men who received CC (*p*=0.450). The two treatment groups exhibited a significant increase in serum total testosterone, while only EC had a statistically significant increase in FSH and LH. Semen volume and concentration did not significantly change with either treatment. Sperm motility increased in both groups, but total motile sperm count (TMSC) only significantly increased in men who received EC.

Conclusions

Our study found that EC and CC are effective treatments in increasing total testosterone without negatively affecting spermatogenesis. EC demonstrated to be more effective in raising gonadotropin levels and TMSC.

## Introduction

Male infertility and hypogonadism are conditions that significantly impact reproductive health and overall well-being in affected individuals [1]. The hypothalamus-pituitary-gonadal (HPG) axis assumes a pivotal role in maintaining male reproductive function, and its intricate interplay becomes crucial in understanding the pathophysiology of hypogonadism. Managing hypogonadism with exogenous testosterone administration may suppress the production of LH and FSH, disrupting the normal hormonal cascade necessary for spermatogenesis [2]. The medical management of hypogonadism and male infertility thus have significant overlap, particularly in the use of HPG-based therapies to stimulate endogenous testosterone and spermatogenesis [3,4].

Clomiphene citrate (CC) and enclomiphene citrate (EC) are medications that are in the armamentarium of those who manage hypogonadism and male infertility. Indeed, CC therapy has been shown to improve sperm concentration, motility, hypogonadism, success rates of surgical sperm retrieval, and potentially spontaneous pregnancy rates [5-7]. In randomized controlled trials of men with secondary hypogonadism receiving EC or topical testosterone, both treatments increased total testosterone (TT), but only EC was found to increase FSH and LH and preserve or increase sperm count [8-11]. The CC and EC function by acting as selective estrogen receptor modulators (SERMs), exerting an agonistic and antagonistic effect on the hypothalamus, respectively. These effects ultimately lead to increased gonadotropin secretion and subsequent stimulation of testosterone production [12].

To our knowledge, there are no clinical studies directly comparing the effects of CC and EC on serum testosterone levels or semen parameters in men. Our study aims to ascertain if either CC or EC has an observable advantage in the management of male infertility and/or hypogonadism.

## Materials and methods

After institutional review board approval, we conducted a retrospective chart review of ≥18-year-old men presenting to the University of Miami with primary infertility, abnormal semen parameters, or hypogonadism who received CC or EC monotherapy between January 2021 and December 2022. Abnormal semen parameters were characterized using the World Health Organization Fifth Edition Criteria [13]. Hypogonadism was defined as serum TT <300 ng/dL with clinical symptoms of hypogonadism, including erectile dysfunction, poor libido, and fatigue [14].

Men were excluded if they were taking concomitant HPG axis-altering therapy, such as human chorionic gonadotropin hormone (HCG) or testosterone. Patients were required to have received at least three months of therapy with EC or CC to be included. We included patients with idiopathic non-obstructive azoospermia (NOA) or NOA associated with anabolic steroid abuse. Individuals with obstructive azoospermia (OA) or NOA stemming from identifiable causes, such as Klinefelter syndrome, Y-chromosome microdeletions, bilateral absence of the vas deferens, cystic fibrosis, Kallmann syndrome, or prolactinoma, were excluded. 

We evaluated body-mass index (BMI), semen parameters (volume, concentration, total motility, and total motile sperm count [TMSC]), and serum hormone levels (total testosterone, FSH, LH, and estradiol) before and after treatment. With a sexual abstinence period of two to three days, all semen samples were analyzed microscopically in the same laboratory by the same andrology technicians. All patients were instructed to obtain bloodwork prior to 10 a.m. We then compared the pre-treatment and post-treatment semen parameters and hormonal levels of each patient.

Statistical analysis

SPSS version 24.0 software was used for statistical analysis. Categorical variables were presented as absolute values and frequencies. For continuous variables, medians, and interquartile ranges (IQR) or means and standard deviations (± SD) were calculated according to the data distribution, as indicated by the normality test. A comparison of the numerical variables between pre-and post-treatment values was performed using a paired t-test.

The differences between post-treatment and pre-treatment mean values were then calculated, and an independent samples t-test was used to determine the relationship between the treatment groups and the difference values. To assess whether changes in hormones and semen parameters after treatment were associated with the BMI and testosterone:estradiol ratio (T/E), analysis of covariance (ANCOVA) was used. ANOVA was also employed to analyze whether changes in hormones and semen parameters after treatment were linked to a past medical history of testosterone abuse. Statistical significance was assessed at p-value <0.05.

As not all hormonal levels and semen parameters were assessed in all of the included subjects, each table incorporates the natural number (N), which denotes the absolute count of individuals who underwent the respective test (sample size). 

## Results

We identified a total of 46 men that received EC and 32 men that received CC who met the inclusion criteria. The median age in years of men on EC was 42 (IQR: 34-47.75), while the median age in years in men on CC was 41 (IQR: 36-44) (p=0.450). There were no differences in baseline BMI, semen parameters, or hormone profile in either of the groups (Table 1). The primary diagnosis in both groups was male infertility (EC: 52.2% and CC: 62.5%).

**Table 1:** Comparison of the Demographic Characteristics in the Clomiphene Citrate and Enclomiphene Citrate Groups. N = sample size (number of participants included); IQR = interquartile range; * = values represented as medians; ^ = values represented as absolute numbers.

Characteristic	Enclomiphene citrate (n=46)	IQR	Clomiphene citrate (n=32)	IQR	p-value
Age (years)	42*	34-47.75	41*	36-44	0.450
Baseline BMI (kg/m^2^)	29.79*	25.06-34.16	32.87*	26.6-34.97	0.060
Primary diagnosis	Enclomiphene citrate (n=46)	(%)	Clomiphene citrate (n=32)	(%)	p-value
Infertility	24^	52.2	20^	62.5	0.172
Hypogonadism	14^	30.4	4^	12.5	
Both	8^	17.4	8^	25	
Anabolic steroid abuse	7^	15.2	5^	15.6	0.966

In the EC group, 14 (30.4%) men received 12.5 mg every day, and 32 (69.6%) men received 25 mg every day. Six (13%) men who received EC were prescribed CC in the past, but stopped treatment due to side effects with the most common being uncontrolled weight gain. No men who received CC had tried EC before. In the CC group, all men received 50 mg every other day.

Treatment with EC resulted in statistically significant increases in TT, FSH, LH, and estradiol following a minimum of three months of treatment. In men who received CC, only TT and estradiol were found to be elevated (Table 2). Eugonadal testosterone levels > 300 ng/dL were achieved in 24 (88.9%) men taking CC and 27 (87.1%) men taking EC. A complete list of hormone parameters following treatment can be found in Table 2.

**Table 2:** Comparative Analysis of the Hormonal Changes Following Three-Month Treatment of Enclomiphene Citrate and Clomiphene Citrate Treatment. N = sample size (number of participants included); IQR = interquartile range. Values represented as medians.

Hormones	Enclomiphene citrate (medians)						Clomiphene citrate (medians)						
	n	Pre-treatment	IQR	Post-treatment	IQR	p-value	n	Pre-treatment	IQR	Post-treatment	IQR	p-value	
Testosterone (ng/dL)	31	390	287.5-561	581	381.5-792	0.026	29	244	165-267	470	301-583	<0.001	
LH (mIU/mL)	20	3.1	1.5-5.1	5.5	3.6-8.4	0.004	24	4.8	3.1-6.8	6.3	3.7-7.9	0.223	
FSH (mIU/mL)	17	4.4	0.8-5.9	7.4	4.3-10.7	0.027	24	6.9	3.8-19.1	13	4.9-19.4	0.070	
Estradiol (pg/mL)	18	29.5	18.7-41.9	67	41.3-108.5	0.003	11	31	14.7-40	37.6	31.3-52	0.074	

Both motility and total motile sperm counts (TMSC) improved in men who received EC, whereas only motility improved following treatment with CC (Table 3). Although sperm concentration increased following treatment in both groups, it did not achieve statistical significance.

**Table 3:** Comparative Analysis of Semen Parameter Changes Following Enclomiphene Citrate and Clomiphene Citrate Treatment. N = sample size (number of participants included); IQR = interquartile range; TMSC = total motile sperm count. Values represented as medians.

Semen parameters	Enclomiphene citrate (n=24) (median)					Clomiphene citrate (n=27) (median)				
	Pre-treatment	IQR	Post-treatment	IQR	p-value	Pre-treatment	IQR	Post-treatment	IQR	p-value
Volume (mL)	2.5	1.5-3.1	1.9	1.2-3.3	0.928	2	1.5-3.4	2.2	1.8-3.4	0.499
Concentration (mill/mL)	1.75	0.6-6.5	8	1.4-13.3	0.152	1.8	0-10.3	8.2	2.3-15	0.087
Motility (%)	23.5	13.5-44.5	42.5	23-55.3	0.043	10	0-46.5	42	24.5-54.5	0.006
TMSC (millions)	0.73	0.20-4.07	4.01	0.7-20.6	0.039	1.2	0-9.6	8	1.6-16	0.262

In men who received EC, before treatment, 75% of the patients had a TMSC < 5 million (n=18), whereas this number dropped to 50% following treatment (n=12). Similarly, in men who received CC, before treatment, 63% of the patients had a TMSC < 5 million (n=17). After treatment, only 37% had a TMSC <5 million (n=10). In both groups, only one patient had a decrease in TMSC from 5-9 million to <5 million (Table 4). Among men who received CC, 22% of the patients were azoospermic pre-treatment (n=seven), from which five (71.4%) patients were found to have sperm in the ejaculate after treatment. Among men who received EC, three patients were azoospermic before treatment, from which two (66.6%) were found to have sperm in the ejaculate following treatment.

**Table 4:** Comparative Analysis of TMSC Distribution Following Enclomiphene Citrate and Clomiphene Citrate Treatment. N = sample size (number of participants included); TMSC = total motile sperm count. Values represented as absolute numbers.

Post-treatment	Pre-treatment						
		Enclomiphene citrate (n=24)			Clomiphene citrate (n=27)		
	TMSC	<5	5-9	>9	<5	5-9	>9
	<5	11 (45.8%)	1 (4.2%)	0	9 (33.3%)	1 (3.7%)	0
	5-9	1 (4.2%)	0	0	2 (7.4%)	0	0
	>9	6 (25%)	0	5 (20.8%)	6 (22.2%)	1 (3.7%)	8 (29.6%)

Prior to treatment, the T/E ratio was 13.2 in men who received EC and 7.9 in men who received CC. After treatment, these values changed to 8.7 and 12.5, respectively. The post-treatment T/E ratio was then classified into two groups: <10 (n=17) and ≥10 (n=13). Subsequently, the after-treatment semen parameters of these two groups were compared. However, no statistically significant differences were observed between the groups (p > 0.05 for all comparisons) (Table 5).

**Table 5:** Comparative Analysis of Semen Parameters Based on Normal Versus Abnormal Testosterone/Estradiol Ratios. N = sample size (number of participants included); T/E = testosterone/estradiol ratio; SD = standard deviation. Values represented as the mean and standard deviation.

Semen parameters	Group T/E< 10 (n = 17) (mean)	SD (±)	Group T/E≥10 (n = 13) (mean)	SD (±)		p-value
Volume (ml)	2.01	1.19	2.83		1.50	0.410
Concentration (mil/ml)	14.26	16.33	12.77		15.09	0.742
Motility (%)	44.58	17.35	33.61		27.28	0.189
TMSC	16.61	21.11	19.18		25.43	0.869

When the pre- and post-treatment mean differences in hormone levels and semen parameters were compared between men who received EC and those who received CC, it was found that no mean difference between the two was statistically significant (Table 6). Baseline BMI was not associated with treatment response to EC and CC for either semen parameter or hormone level (p>0.05 in all).

**Table 6:** Comparative Analysis of the Mean Differences in Hormonal Levels and Semen Parameters Following Enclomiphene Citrate and Clomiphene Citrate Treatment. LH = luteinizing hormone; FSH = follicle-stimulating hormone; TMSC = total motile sperm count. Values represented as mean differences.

Variable	Enclomiphene citrate (mean difference)	Clomiphene citrate (mean difference)	p-value
Hormones			
Testosterone (ng/dL)	124.96	177.30	0.491
LH (mIU/mL)	3.54	1.02	0.067
FSH (mIU/mL)	4.42	8.47	0.765
Estradiol (pg/mL)	61.18	25.01	0.164
Semen parameters			
Volume (mL)	0.02	-0.17	0.578
Concentration (mill/mL)	4.98	4.73	0.953
Motility (%)	0.08	0.14	0.198
TMSC	20	27	0.676

## Discussion

In men with low testosterone who desire fertility, finding alternatives to testosterone replacement therapy (TRT) that increases the total and intratesticular testosterone levels while preserving spermatogenesis is imperative. Off-label medications used for this indication include SERMs, gonadotropins, partial estrogen agonists, and aromatase inhibitors [15,16]. The most extensively studied and accepted SERM in this patient population is CC [17]. Recently, EC, the short-acting isomer of CC, has been reported to offer a better safety profile [18]. Identification of the enhanced efficacy of EC brings attention to the benefits of prescribing this medication to men with low testosterone and infertility. In the limited current literature for EC, the most prevalent weaknesses are small sample sizes and a paucity of comparisons between EC and other SERMs [19]. This study aimed to directly compare the efficacy of EC to CC in terms of improving various sperm parameters and hormone levels in men with hypogonadism and infertility.

Within our cohort, both CC and EC were successful in improving serum testosterone levels. Krzastek et al. reported similar results with the use of CC in patients who initially were hypogonadal [20]. Similarly, a clinical trial that compared EC and 1% topical testosterone found that EC successfully increased the morning levels of TT to a mean of 525 ± 256 ng/dL [8].

Although not Food and Drug Administration-approved for hypogonadism, the American Urological Association and European Association of Urology support the use of CC as an off-label therapy for increasing testosterone levels in men who desire to maintain fertility [15,16,21]. In the case of CC, its impact on gonadotropins is controversial. We found that FSH and LH were not significantly changed after treatment; however, other studies have shown CC increases FSH and LH, while some showed only an improvement in LH [20,21].

Post-treatment levels of estradiol increased in both the EC and CC groups, although it was only statistically significant in the EC cohort. The scarce literature evaluating the side effects of EC and CC highlights that both medications have the potential to significantly increase estradiol levels [19,20,22,23]. Theoretically, EC has been proposed to cause less estrogenic effects than CC, as it has been said that it is the cis-isomer, and not the trans-isomer, of CC that causes the rise in estradiol [24]. However, our results showed that the T/E ratio considerably decreased in men who received EC, while it significantly improved in men who received CC. Similar results were seen in a study of 36 men with hypogonadism treated with a daily dose of 25 mg CC. The T/E ratio after six weeks of treatment increased from 8.7 to 14.2 (p< 0.001) [25]. Unfortunately, there are no current studies analyzing the T/E ratio after treatment with EC, which identifies the need for further studies with EC and T/E ratio to better understand their relationship.

The role of estradiol in spermatogenesis is still a matter of debate. A meta-analysis of 666 men who received either letrozole or anastrozole due to high estradiol levels found that after treatment, FSH, LH, testosterone, and sperm concentration improved significantly [26]. However, motility was negatively impacted by aromatase inhibitor therapy, leading the authors to recommend a one-week cessation of medical treatment prior to sperm retrieval [26]. In our study, we conducted a subset analysis comparing post-treatment semen parameters and hormone levels in men with T/E ratios of <10 versus ≥10, but we did not find any significant differences between the two groups. Further and larger studies are needed to better understand the role of estradiol and its optimal level for promoting spermatogenesis.

In terms of semen parameters, CC improved only sperm motility in our study. This contrasts with results found in some investigations where CC increases sperm count, motility, concentration, volume, or density [21,27]; however, other studies found no significant improvements in semen parameters for men using CC, which demonstrates the relative inconsistency of its benefits and the need to identify a subset of this population who are better candidates for this medication [21]. In the case of EC, it demonstrated a significant increase not only in motility but also in the TMSC. Sperm motility has been shown to be preserved with EC treatment [9], but this is the first time an improvement in both motility and TMSC is being reported. 

The global prevalence of anabolic-androgen-steroid abuse among men is reported to be 6.4% [28]. Exogenous testosterone has been shown to inhibit spermatogenesis and intratesticular sperm production [28,29]. Off-label use of HCG and CC have been employed as recovery therapies for men taking testosterone, and studies have demonstrated that they can reduce the recovery time of sexual hormones and sperm parameters [30]. It has been reported that, after cessation of androgen abuse, about 90% of men can recover their fertility potential [29]. In our cohort, 12 men had a history of anabolic steroid abuse. Post-treatment semen parameters and hormone levels were not significantly different between men with and without previous testosterone use, except for estradiol, which was found to be significantly higher in men with a history of anabolic steroid use. This is the first study showing that EC may be used as a therapeutic option for spermatogenesis and sexual hormone recovery in men who have previously used anabolic steroids.

Our study is not without limitations. As a retrospective, single-institution study with a modest sample size, the potential for both selection and ascertainment bias is possible. Those who received EC had a higher baseline testosterone level compared to those who received CC and a higher number of patients were azoospermic in the CC group compared to the EC group, which may have impacted the results seen in our study. Moreover, due to the small sample size, we were unable to age-match patients between those who received CC and EC. While this was a limited cohort of patients, we believe that the result of our study identifies the need for future studies analyzing the use of EC. We expect that a prospective study with a longer follow-up and groups matched to help exclude possible confounding factors would yield more substantial evidence to support the increased use of EC in clinical settings.

As male infertility carries a substantial social, psychological, and economic burden, various treatment modalities must be explored to determine how to help this population without the added stress that TRT carries. Our study provides promising results for the use of EC and CC and further emphasizes the importance of additional research on treatment options for male infertility to ensure patients receive the best therapy with established long-term safety and efficacy.

## Conclusions

Both EC and CC resulted in improvements in serum testosterone levels, though estradiol was significantly increased in men receiving EC compared with CC. The clinical relevance of this is unclear. Both both EC and CC therapy resulted in improved sperm motility, while only EC showed an added benefit of improving TMSC. Both medications also showed promise in elevating TMSC levels to offer them the chance of exploring intrauterine insemination and natural conception. Although this direct comparison between EC and CC provides insight into the benefits that EC may offer these patients, further research is needed to support our findings.
